# Cellularized small-caliber tissue-engineered vascular grafts: looking for the ultimate gold standard

**DOI:** 10.1038/s41536-021-00155-x

**Published:** 2021-08-12

**Authors:** Adrien Fayon, Patrick Menu, Reine El Omar

**Affiliations:** 1 Université de Lorraine, CNRS, IMoPA, F-54000 Nancy, France; 2 Université de Lorraine, Faculté de Pharmacie, Nancy, F-54000 France

**Keywords:** Cardiovascular diseases, Tissue engineering

## Abstract

Due to the lack of efficacy of synthetic vascular substitutes in the replacement of small-caliber arteries, vascular tissue engineering (VTE) has emerged as a promising solution to produce viable small-caliber tissue-engineered vascular grafts (TEVG). Previous studies have shown the importance of a cellular intimal layer at the luminal surface of TEVG to prevent thrombotic events. However, the cellularization of a TEVG seems to be a critical approach to consider in the development of a TEVG. To date, no standard cellularization method or cell type has been established to create the ideal TEVG by promoting its long-term patency and function. In this review, advances in VTE are described and discussed with a particular focus on the construction approaches of cellularized small-caliber TEVGs, the cell types used, as well as their preclinical and clinical applications.

## Introduction

Cardiovascular diseases (CVD) are the major cause of death worldwide^1,2^, representing 32% of all global deaths^3^. These diseases are often associated with a dysfunctional vasculature caused by inflammatory, metabolic and proliferative alterations leading to the narrowing or complete obstruction of blood vessels. Thus, tissue damage is induced by diseased blood vessels that are unable to convey oxygen and essential nutrients to cells. Common presentations of CVD are coronary heart disease, peripheral arterial disease, deep vein thrombosis, and cerebrovascular disease^4^.

Consequently, CVD have resulted in an increasing need for vascular grafts in order to reconstruct or bypass vascular stenosis. Vascular bypass grafting remains one of the most effective treatment approaches for patients who need long-term revascularization^5,6^. For the case of myocardial revascularization, coronary artery bypass grafting (CABG) remains the most common cardiac surgery procedure in the world, with about 200,000 CABGs performed annually in the US, while in Western European countries an average incidence of 62 per 100,000 people is reported^7^. It is worth mentioning that vascular conduits are also required to create arterial–venous fistulas for frequent hemodialysis access.

Although autologous arteries and veins are the preferable conduits for vascular grafting, their use may be impossible due to their limited availability, their variable quality (e.g., varicose veins), and the complications associated with their removal (i.e., infections, tissue damage, time consuming procedures, and long-term recovery). As an alternative to autografts, synthetic vascular grafts can be used as they have shown a long-term efficacy in the replacement of large—(>8 mm) and medium—(6–8 mm) diameter arteries. However, they present poor patency rates when used for bypassing small-diameter arteries (<6 mm)^4^.

Globally, small vascular graft failures are commonly due to intimal hyperplasia, thrombosis, atherosclerosis, or even through infections. Indeed, one of their major limitations is the absence of their luminal cellularization, leading to thrombosis via the adherence of blood proteins and the activation of clotting mechanisms^8,9^.

The limitations of small-diameter vascular grafts are a real obstacle to the vascular bypass substitutes, making this a real public health issue^4,10^. The production of a tissue-engineered vascular graft (TEVG) for the replacement of small-diameter vessels has become the most heavily investigated and challenging areas of vascular tissue engineering (VTE). A TEVG with long-term patency, capable of being remodeled by the host organism, and showing self-repair capacities in vivo would be of great benefit^11^. Such a TEVG may represent a major advance in the field of cardiovascular surgery by providing an allogeneic “off-the-self” solution for clinical use allowing a new therapeutic option in various pathological states such as myocardial and lower limbs revascularization, pediatric vascular disease, useful also as arteriovenous bridges for hemodialysis. Furthermore, an “off-the-shelf” TEVG will be perfectly adapted for mini-invasive surgeries performed by robots. In fact, surgical robotic technology has emerged to perform remote operations in limited spaces and activities and is considered as the surgically invasive version of coronary bypass, avoiding a complete thoracotomy or sternotomy, thus preserving the thoracic integrity and suffering of the patient, and significantly reducing hospitalization period and costs.

The first TEVG developed was reported by Weinberg and Bell in 1986. It was produced by mimicking the blood vessel architecture using bovine aortic endothelial cells (ECs), smooth muscle cells (SMCs), thin Dacron^®^ mesh, and collagen to create a tubular scaffold. On this scaffold, ECs, SMCs, and bovine adventitial fibroblasts were co-seeded on the luminal surface, the internal wall and on the external side, respectively^12^.

**Fig. 1:**
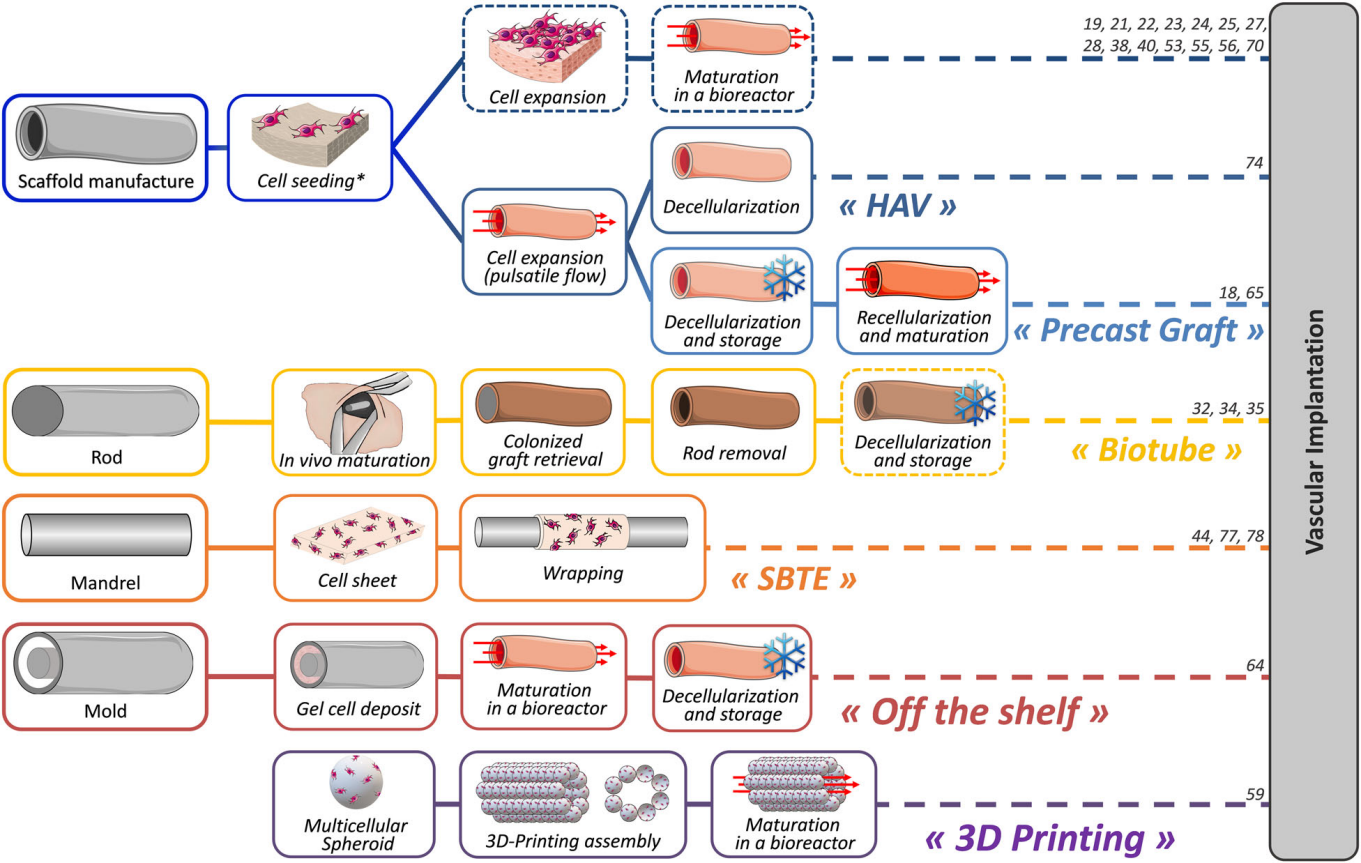
Fabrication approaches to produce a small-diameter TEVG. Dotted frames represent optional steps found in the literature. *Cells can be seeded using cellularized gel or cell suspension. TEVG Tissue-Engineered Vascular Graft, HAV Human Acellular Vessel, SBTE Sheet-Based Tissue Engineering.

Since this first TEVG, several small-diameter cellularized vessel substitutes have been elaborated and tested with different production approaches which are presented in Fig. 1.

A variety of scaffolds from natural sources can be used, such as decellularized vessels or in vitro developed scaffolds, e.g., synthetic or biosynthetic grafts. Associations of different cell sources and different combinations of microenvironmental conditions to lead cell differentiation or maintain cell integrity have also been described. Despite these considerable advances in VTE, only few grafts have reached clinical studies and the perfect TEVG has not been yet developed.

A key factor to consider in the development of a TEVG is the cellular component. In fact, a very recent meta-analysis of preclinical studies using small-diameter TEVG has found that recellularized grafts were more patent than acellular grafts (85.1% vs. 64.4% respectively)^13^.

The cell adhesion, proliferation, as well as differentiation and several other contributions to hemostasis is crucial to create a hemocompatible small-diameter vascular graft. In physiological conditions, the blood vessel hemocompatibility is ensured by a monolayer of ECs localized at its luminal surface (termed the “endothelium”). This blood contact surface maintains hemostasis through several antithrombogenic mechanisms, such as the synthesis of plasminogen activator (tPA); inhibition of platelet aggregation via secretion of nitric oxide (NO) and prostacyclin; and by the presence of glycosaminoglycans on the endothelial cellular surface preventing blood coagulation^14,15^. Thus, in order to ensure optimal hemostasis, a continuous cell layer on the luminal surface of the vascular substitute is required. For this purpose, different cellularization approaches are described in the literature, using variable amounts of cells according to the seeding technique and the maturation period of the graft. The cell adhesion and retention can be highly improved by a prior coating deposit on the luminal surface of the graft. The addition of a luminal coating in the TEVG manufacture protocol is highly recommended as it can modify the microenvironment and guide cell behavior by promoting cell adhesion, proliferation and/or differentiation^16^. Several types of coatings have been used, composed of one or a combination of proteins which are in most cases of animal origin. Our team has recently developed an innovative approach to coating surfaces using a natural extracellular matrix (ECM) of human origin, extracted from the umbilical cord Wharton’s jelly (WJ) and is totally natural, biocompatible, and perfectly controllable^17^.

This review will focus on the cellularized vascular grafts described in the literature, the different manufacturing protocols, the various quality control tests, the preclinical in vivo evaluation in several animal models and different implantation sites, as well as the few human clinical trials. The results will be presented and discussed critically, with an emphasis on the weak points for each TEVG and the proposition of solutions for their improvement.

## Preclinical trials

Several approaches have been described for the cellularization of TEVG using different types of cells in order to recreate the intimal and medial layers. Cells used in VTE can be from autologous or heterologous origins, as discussed in the following paragraphs of this review.

### Substitutes using autologous cells

The advantage of using autologous cells is the avoidance of any host immunological reaction which induces the graft rejection. Two cellularization approaches have already been reported in the literature: (1) an in vitro method involving the harvesting of autologous animal cells before the preparation of the substitute and (2) an in vivo method which uses the animal model as an “incubator” by placing an acellular tube in the animal before vascular grafting. These studies are part of a proof-of-concept approach; they propose TEVG manufactured using autologous animal cells that are not directly transferrable to human implantations. Moreover, the use of this cell type requires fairly long preparation times of up to several months^18^, which may be incompatible with the critical situation of the patient.

#### Endothelial cells (ECs) and endothelial progenitor cells (EPCs)

Ma et al. have reported the use of mature autologous canine ECs in the manufacture of their graft^19^. Cells were harvested from the left external jugular vein. Cells were cultured until the 4th passage and then seeded by rotational precipitation into a decellularized aortae of fetal pigs, clamped on both sides (arteries are rotated 120° every 10 min for 30 min). After the EC deposit the graft was placed in a bioreactor for 7 days, allowing a step of dynamic culture where the perfusion rate was increased from 10 to 60 mL/min, followed by 3 days of static culture. The autologous graft was then implanted as a carotid graft replacement in seven dogs for a period of up to 6 months. Results showed an absence of thrombi and stenosis and all the grafts remained patent over the period of the study. After 6 months, the intimal layer of the graft was composed by EPCs and ECs whose origin has not been precisely determined. Moreover, the graft was remodeled by host cells, notably muscular fibroblasts which settled in a “media-like” layer. Although the graft has shown satisfactory results, some issues could be addressed further, especially in regards to the animal model used in the study. Thrombogenesis mechanisms and vascular viscoelastic properties in dogs are very different from the human physiological parameters^20^. Thus, these results cannot necessarily be transferrable to humans.

ECs can also be obtained from EPCs that are collected non-invasively from the host’s peripheral blood. These cells can be used directly to cellularize a scaffold or can be subjected to a prior differentiation step before being seeded.

In 2001, Kaushal et al. evaluated the in vivo efficiency of a TEVG built from a pig decellularized iliac artery in a sheep model. The artery was seeded with autologous EPCs harvested from the internal jugular vein of 1 to 2 week(s) old sheep^21^. Seeding was accomplished under dynamic rotating conditions for 6 h. The graft was then matured in a laminar flow bioreactor for 6 days with a gradually increasing shear stress and implanted in an end-to-end carotid artery replacement model. Two groups of animals were compared under the same grafting conditions: seven animals receiving seeded grafts *vs*. four animals with unseeded grafts. The grafts patency was monitored by ultrasound Doppler every day, for a period ranging from 15 to 130 days. In the first 5 days of implantation, three of the four unseeded grafts were 100% clogged. The 4th graft was 50% clogged 15 days after the implantation. Concurrently, all seeded grafts remained patent over the entire study period (*n* = 3 at 15 days; *n* = 4 at 130 days). At 15 days, a layer of ECs derived from originally seeded EPCs was present, however the media of the substitute remained acellular. At 130 days, the results showed a thickening of the media that was infiltrated by host cells void of smooth muscle alpha-actin (α-SMA) expression, as well as an intimal layer colonized by α-SMA+ cells. Only 10% of the ECs identified at the luminal surface at 130 days were derived from the originally seeded EPCs. After 130 days, collected grafts showed a vasoconstriction capacity following exposure to serotonin and norepinephrine, reflecting the functionality of SMCs found in the intimal layer. On the other hand, these same grafts showed a relaxing capacity probably mediated by the release of NO, similar to that found in the native carotid artery.

Otherwise, EPCs can be subjected to a prior differentiation step before seeding, as achieved by Tillman et al. in 2012^22^. In their study, the substitute was developed by seeding differentiated autologous circulating EPCs on a decellularized porcine carotid artery segment. The seeding was carried out in a bioreactor by injecting the cells into the lumen of the vessels for 2 h. The graft was then subjected to a gradually increasing nonpulsatile flow for 5 days. Graft maturation was then completed after 9 days under pulsatile flow. This substitute was evaluated for arteriovenous flow and needle punctures in the ovine model. “Short-term” and “long-term” groups were monitored. The “short-term” group, consisting of a study period of 2 months, included seven grafted animals. Grafts of this group were punctured 3 times a week during the second month. In the “long-term” group, eight animals were implanted and followed for 6 months. From the 5th month, grafts were punctured 3 times a week. During this experiment, of the total number of grafts, four were stunted in the first month of transplantation (the healing month) and occlusions appeared from 3.3 months. This type of graft using decellularized blood vessels as scaffolds have shown resistance deficiencies to repeated punctures. Other scaffolds, such as those built by mesh construction could be more effective in needle resistance.

#### Combination of EPCs and SMCs

Previous studies have described a co-seeding method with a combined cellularization, using autologous ECs and SMCs to improve the TEVG resistance. This approach was evaluated by Neff et al. in a carotid artery interposition grafting model in pigs for 4 months^23^. Results were compared with those obtained from TEVG seeded only with ECs. The manufacture of this graft consisted of the decellularization of a pig carotid artery, followed by an incubation with a SMC suspension under rotation into an outer chamber. Then, the graft was placed in a bioreactor where the lumen was perfused with a suspension of ECs for 10 to 14 days. Results have shown that the addition of SMCs to the graft wall allowed a better cellularization of the graft after implantation. This method of combined cellularization also reinforced the TEVG contractility without a negative impact on its permeability. However, this contraction capacity was much weaker than that of a native carotid artery (15–20%).

Koch et al.^24^ and Ju et al.^25^ have applied the co-seeding technique to a fibrin scaffold supported by a poly(L/D)lactide 96/4 [P(L/D)LA 96/4)] mesh and an electrospun bilayered vascular scaffold, respectively. The same cellularization protocol as previously described was utilized by Ju et al. Koch et al. produced their scaffold by a SMC/fibroblasts/fibrinogen suspension that was left to polymerize inside a mold. After polymerization, the luminal surface was cellularized with an ECs suspension into a rotating chamber^26^. Both TEVG were evaluated in a sheep carotid arterial interposition model. After an implantation period of 6 months, promising results have shown that the TEVG remained patent and well-integrated into the host tissue. Although these grafts appear to be suitable for clinical applications, some issues must be addressed, such as the long processing time. In fact, the scaffold preparation requires 7 days, followed by several weeks of autologous cell expansion until the 3rd–5th passage. An evaluation of this graft in a CABG or peripheric vascular bypass model would be beneficial to validate its efficiency in different representative clinical situations.

#### Modified cells

Another approach to obtain suitable efficient TEVGs is to modify seeded cells to confer them an additional functionality or to enhance a specific function. McIlhenny et al. have proposed a TEVG consisting of a decellularized human saphenous vein seeded with rabbit autologous adipose derived stem cells (ASCs). Cells were obtained from dorsal fat pads and seeded by a rotating perfusion of the graft’s luminal surface. The cellularized graft was then matured for 1 week in a bioreactor with a linearly increasing shear stress rate, thereby inducing the ASCs differentiation toward endothelial-like cells (ELCs)^27^. A first implantation attempt showed a high thrombogenicity due to a weak production of NO by ELCs. Therefore, in order to increase the NO production by ELCs, a transfection of ASC was achieved with an adenoviral vector containing the endothelial nitric oxide synthase (eNOS)^28^. The TEVG with modified cells was tested in five rabbits in an abdominal aortic interposition graft model. The control group consisted of five rabbits implanted with unseeded grafts. After 2 months of implantation, all grafts, seeded and unseeded, had remained patent. Histological analyses have demonstrated that unseeded grafts exhibited a thrombus formation and/or a thrombin and fibrin staining at their luminal surface. Seeded grafts on the other hand, presented a smooth luminal surface without any traces of thrombin or fibrin. Although transfected cells have allowed the improvement of the graft characteristics via the overexpression of eNOS, the long-term patency and efficiency need to be evaluated in larger animal models which have a more similar physiology to humans. Moreover, the originality of this graft seems to be a major default since gene editing for clinical applications is at the center of ethical debates^29^.

#### Graft production in situ by host cells

Grafts developed according to this method are called “biotubes” and are derived from the “in-body tissue architecture technology”. The preparation consists of a natural or synthetic rod implanted subcutaneously into the host tissue and left to be colonized/encapsulated by host cells. After the in vivo incubation period, the encapsulated rod is harvested. The rod is then removed, and the host tissue surrounding the rod is used as a TEVG. Different kinds of tubes were used in this context, such as a PGA tube^30^, poly(methyl methacrylate) (PMMA) tube^31^, poly(vinyl chloride) (PVS)^31^, etc. Watanabe et al.^32^ developed a biotube by subcutaneously implanting a silicone rod of 3 mm diameter into rabbits for 2 months. In their previous study, the resultant biotube was described as an autologous connective tissue of 76 ± 37 µm of thickness, composed of collagen and fibroblasts^33^. This biotube was then evaluated in a carotid implantation model in the same rabbit, for 26 months. Histomorphological analyses of explanted grafts showed the absence of neointimal thickening. The luminal surface was entirely covered with PECAM-1 positive ECs, under which α-SMA positive cells and elastic fibers were found. Also, these α-SMA positive cells and collagen were remarkably oriented. Although these results seem promising, this study focused more on the manufacturing protocol than on the arterial implantation, since the latter was achieved in only one rabbit. Results need to be confirmed in a larger study including more animals with a control group. In 2016, the same fabrication approach was performed in vivo by Rothuizen et al. in pigs^34^. Rods were subcutaneously implanted for 4 weeks and harvested with the surrounding tissue. The biotubes produced were principally composed of collagen (type I and III) and glycosaminoglycans. Despite an important graft wall colonization mostly by fibroblasts and leukocytes, no endothelium-like layer was observed. 8 TEVGs have been studied in 4 female Landrace pigs (bilateral carotid artery interposition) for 4 weeks. After 4 weeks of vascular integration, 7/8 grafts were still patent. The described occlusion was presumably due to a pre-anastomotic intimal hyperplasia which was absent in the contralateral graft. Histological analyses of the integrated graft have shown an increase in the wall thickness and an acquisition of a contractile phenotype by fibroblasts. Interestingly, a confluent layer of lectin positive cells on the graft luminal surface was present, which may suggest a graft endothelialization had occured during vascular integration. However, the major inconvenience of this “biotube” technique remains the long in vivo maturation period and the complicated transposition for clinical application. To overcome these limits, very recently, Yamanami et al.^35^ used the same principle by testing biotube cellularization in Beagle dogs for 4 weeks. After explantation, biotubes were decellularized and stored at −20 °C for 1 week. Before implantation, decellularized grafts were treated with a direct thrombin inhibitor—argatroban, to confer antithrombogenic properties to the graft. Grafts were then implanted at the infrarenal abdominal aorta in three adult male Wistar rats. At 1-month post implantation, grafts were still patent, and seemed to be infiltrated by host cells. However, the tested grafts were only 1 cm long. To meet clinical needs, longer grafts should be tested in larger animal models and in different implantation sites to evaluate their patency over long periods.

### Substitutes using allogeneic cells

In order to address the issue of small-diameter vascular substitutes, numerous studies have included allogeneic cells in the fabrication protocol of TEVG. The main advantage of these cells is the possibility of obtaining a “ready-to-use” cellularized graft. However, two limitations arise: (1) the host immunological reaction towards foreign cells and (2) the storage conditions of cellularized grafts before their use. Different solutions are proposed to overcome these obstacles and to produce readily available “universal” small vascular grafts. Various models are described in the literature, such as grafts manufactured directly using modified or unmodified allogeneic cells, or using several cell types of mixed allogeneic and autologous sources. Moreover, some authors are trying to provide an “off the shelf” graft by promoting allogeneic cells to produce their own ECM that will constitute the graft’s scaffold. The latter will be subjected to a decellularization step and can be recellularized with patient autologous cells just before the implantation. This approach provides a “personalized” cellularized graft for the patient^18^. Production methods described in this section will be divided into conventional and innovative production techniques.

Conventional TEVGs production approaches using allogeneic cells can be produced directly by a single “simple cellularization”. Several cell types have been evaluated, such as bone-marrow mononuclear cells (BMNCs), EPCs, SMCs, ECs, and induced Pluripotent Stem Cells (iPSCs)^36^. Modified cells were also used in order to enhance the proliferation capacity of allogeneic cells (which decline with the donor age) or to confer antithrombogenic properties to the used cells.

#### Bone-marrow mononuclear cells (BMMCs)

The BMMCs population is known to have a differentiation potential in both endothelial and SMCs, meaning they are widely used in VTE^37^. To create their own small-diameter vascular graft, Roh et al.^38^ used commercial human BMMCs (hBMMCs) that were suspended into a fibrin gel solution and seeded onto a biodegradable Polyglycolic Acid–Poly-**L**-Lactide and -ε-Caprolactone (PGA-P(CL/LA)) scaffold (with an internal diameter of 0.7 mm and a wall thickness of 250 μm)^39^. The in vivo study consisted of an inferior vena cava interposition into 3–4-month-old female SCID/bg mice for up to 24 weeks. At the end of the study, 9 of 10 seeded grafts were still patent. Histological analysis has shown an endothelial cell layer at the luminal surface and two layers of smooth vascular cells. However, cells composing the graft were exclusively from murine origin. Authors have suggested that the graft colonization by host cells was mediated by MCP-1 (monocyte chemoattractant protein-1) released by hBMMCs. The MCP-1 release led to monocyte infiltration and to inflammatory mechanisms involved in blood vessel formation and remodeling. Regarding the immunodeficient small animal model used, results do not allow the evaluation of the grafts immunological state. Indeed, several extensive studies are required to assess the host immune response triggered towards the graft. Furthermore, hBMMCs have not shown expected results concerning their differentiation potential into vascular cells in order to remodel the graft. This may be due to the inappropriate animal model used, lacking immunomodulatory cells that may be involved in blood vessel remodeling.

In an another study, Fukunishi et al.^40^ tested a PGA-PLCL TEVG (inner diameter of 1 mm) in an inferior vena cava interposition model in C57BL/6 mice for 6 months. Seeded grafts were studied in comparison with unseeded grafts to determine the role of BMMCs. Grafts were seeded with allogeneic BMMCs and harvested from donor mice, followed by a perfusion of the cell suspension into the scaffold lumen for 10 min. Seeded grafts were then incubated overnight at 37 °C prior to the implantation. At the end of the study, 9 of 10 seeded grafts remained patent. Moreover, this study demonstrated that the BMMCs seeding prevented the graft narrowing and stenosis that were observed in unseeded grafts. This phenomenon seems to be mediated by a macrophage modulation and a prevention of platelet activation, induced by seeded BMMCs. This study complements other studies that have reached clinical trials for arteriovenous grafts with an inner diameter of 10 mm and could provide a new cellularization approach for next generation grafts.

#### Mesenchymal stem cells (MSCs)

In addition to their differentiation potential into vascular phenotypes, MSCs exhibit important immunomodulatory capacities, such as T cell^41,42^, the generation of a regulatory T-cell population and the modulation of Natural Killer cells (NK) and macrophage responses^43^. In order to demonstrate the antithrombogenic potential of MSCs, Hashi et al.^44^ produced a cellularized small TEVG by seeding human bone-marrow MSCs on an electrospun nanofibrous membrane for 1 day. This cellularized membrane was then rolled and sutured around a 0.7 mm diameter mandrel. After removal of the mandrel, the graft was kept in culture for 2 days and then implanted for up to 60 days as a common carotid artery replacement in 6–8 week athymic rats. Results suggested that MSCs can modulate the host inflammatory response by preventing platelet adhesion and aggregation. These cells also showed antithrombogenic capacities by decreasing platelet aggregation (similar to ECs capacity) compared to unseeded graft controls. This was supported by a low and stable intimal thickening in seeded grafts 1-month post implantation, whereas a significantly higher thickening of the vascular wall was observed in unseeded implanted grafts. Both unseeded and seeded grafts showed an efficient recruitment of host ECs and SMCs which led to an organized layered vascular wall similar to a native artery. The data seems to demonstrate a short-term engraftment activity and efficacy of MSCs which are rarely found 2 months post implantation. MSCs appear to have beneficial effects, making them a promising cell source to consider in VTE. MSCs seeded grafts should be further evaluated under more challenging conditions, with immunocompetent larger animal models, longer implantation periods and at different implantation sites. Moreover, a more in-depth analysis regarding the fate of MSCs in the graft would also be interesting in order to understand their specific effects on the vascular wall remodeling, the recruitment of host cells, the modulation of the inflammation induced by the graft and their in vivo differentiation potential.

Inspired by the human coronary artery structure, a study has described a TEVG produced by an innovative approach, involving the deposition of layers of cell-laden hydrogel, by combining a dip-spinning technology with a solution blow spinning device (dip-spinning-SBS technology) within 30 min^45^. The substitute wall was composed of BM-MSC cellularized gelatin hydrogels and PCL fibers which were automatedly arranged to reproduce the J-shaped mechanical response and compliance of human coronary arteries. Preliminary in vivo tests were conducted in six rabbits, as a carotid replacement model, divided into three groups: animals receiving cellularized grafts, animals receiving acellularized grafts and a control group consisting of an incision-anastomosis. Histological studies were performed at 14 days and 30 days post implantation to particularly observe the graft remodeling. Although a thrombus development was observed in both grafts (cellularized and acellularized), complete vascular wall remodeling was achieved at only 30 days in cellularized grafts. This study encourages the consideration of MSCs for TEVG cellularization as they prevent inflammation and promote graft remodeling after implantation.

MSCs seem to be a promising cell source for VTE^46^. These cells are preferentially harvested from bone marrow, but they are also found abundantly in many other tissues such as dental pulp, adipose tissues, or perinatal tissues. For VTE, MSCs derived from perinatal sources, such as the WJ of the umbilical cord, may be an appropriate cell type for VTE applications. Wharton’s jelly derived MSCs (WJ-MSCs) have all the characteristics of MSCs, possess a more primitive phenotype than other mature MSCs and their use is totally safe as they do not induce teratoma formation^47^. They were shown to differentiate toward vascular phenotypes^48–50^. Moreover, for VTE purposes, our team has already shown that WJ-MSCs subjected to an endothelial differentiation, conserved their immumodulatory properties after differentiation and remained immunosuppressive by inhibiting the proliferation of T cells, reducing the toxicity of NK cells and generating a population of regulatory T cells^51^.

#### Muscle derived stem cells (MDSCs)

MDSCs have also been integrated in the cellularization protocol of some TEVGs. MDSCs are known for their high proliferation rates, a low expression of major histocompatibility complex class I and their in vivo and in vitro multilineage differentiation potentials toward muscle, neural, and ECs^52^. Nieponice et al.^53^ compared three different TEVGs: (1) unseeded Poly(ester urethane)urea biodegradable tubular scaffolds produced by thermally induced phase separation (unseeded TIPS-PEUU), (2) TIPSS PEUU seeded with MDSCs (TIPS-PEUU), and (3) a MDSC seeded graft manufactured by combining TIPS with an electrospinning technique (ES-TIPS-PEUU). Autologous MDSCs were isolated, labeled by transfection with the LacZ reporter gene (to allow their postimplantation detection) and amplified, before being seeded on grafts of 15 mm length and 1.5 mm inner diameter, with a rotational vacuum seeding device^54^. Grafts were then cultured for 48 h under agitation. After 8 weeks of implantation in a rat aorta interposition model in Lewis rats, results reported that 73% of seeded TIPS-PEUU grafts showed signs of stenosis or aneurysmal dilatations and 65% of ES-TIPS-PEUU were patent without aneurysms, whereas only 10% of unseeded TIPS-PEUU grafts remained patent. Grafts were colonized by host cells, with von Willebrand factor positive cells at the luminal surface and cells with a contractile phenotype in the graft tissue. Moreover, a few transfected cells were still found in the grafts. Thus, the ES-TIPS-PEUU construct produced by double processing techniques could provide a promising solution for VTE. However, further analyses are required in larger animal models with an adaptation of the construction protocol to meet the clinical requirements.

#### iPSCs

iPSCs are among cells that have been investigated by several teams for the cellularization of small-caliber TEVG. Hibino et al.^55^ have used commercial male mouse iPSCs that were differentiated into sheets of ECs and SMCs, before being seeded onto PGA-P(CL/LA) with a diameter of 0.8 mm. This graft was implanted as an infrarenal inferior vena cava in female SCID/bg mice. After 10 weeks of implantation, all grafts were still patent with no signs of aneurysms or calcification. Immunohistological analysis of collected grafts revealed the gradual loss of the originally seeded cells and their replacement by host ECs at their luminal surface and SMCs at the inner layer. Wang et al.^56^ have also described an in vivo study where the TEVG were cellularized with iPSCs derived from primary human aortic fibroblasts which differentiated into proliferative SMCs. The cell seeding was carried out by an incubation of 24 h on PLLA scaffold. The vascular graft was placed in the subcutaneous pocket into nude mice for up to 2 weeks. This study has not evaluated the graft capacities and function but has explored an original approach, allowing the obtention of a personalized TEVG using the patient autologous cells.

Although iPSCs seem to be a very promising source for new therapeutic strategies and regenerative medicine, due to the major ethical concerns around the use of embryonic stem cells, their use is still debated. Human iPSCs are produced with poor efficiency, moreover, the reprogramming protocols involve viral vectors that lead to safety issues for clinical applications^48^. In addition, even after differentiation, a residual presence of undifferentiated cells represents a high risk of tumorigenicity that may lead to the development of teratomas and malignant tumors, which affects their safety for human medical applications^57^.

#### Multicell spheroid (MultiCS)

Bio-printing is a biomedical application using the principles of 3D printing to artificially produce living biological tissue. Bio-printing is a fast-growing field today because its potential applications are vast and promising in the medical field as well as in research^58^. Itoh et al. have used this technology to produce a TEVG in 4 days by assembling 500 multicell spheroids as a tube of 1.5 mm inner diameter and 7 mm in length^59^. These MultiCS (size of 615 ± 51.3 µm) were randomly composed of commercial human umbilical vein ECs (40%), human aortic SMCs (10%), and human dermal fibroblasts (50%). The tubular construct was matured in a bioreactor with a triple medium mix for 4 days under an increasing perfusion rate. It was then integrated in an abdominal aorta replacement model in nude rats for a period of 2 to 5 days. At the end of the study, ECs that were originally integrated into MultiCS were found at the luminal surface of the graft, forming an intima-like layer. Interestingly, an increase of the graft inner diameter was also observed. Although the immunodeficient model is essential to avoid immune rejection when using mature adult cells, it did not allow investigations into TEVG safety and the interactions between MultiCS and the host immune cells, in addition to the potential modulation of the host inflammatory response. Moreover, the graft length was only 7 mm which is incompatible with clinical applications (bypasses, vessel replacement, arteriovenous fistula, etc.,). As already mentioned for other studies, this graft should be adapted for clinical use regarding its length and the origin of the integrated cells. A deeper characterization should be conducted around its safety, its patency and efficiency.

In the VTE field, allogeneic cells are widely studied. In fact, the cell type is an essential and determining criterion in the construction of TEVG with regard to the required functionality. TEVG with mature ECs and SMCs are still in the preliminary study phases, in which nude animals are used to allow the observation of the graft development once integrated into the blood circulation. However, several problems inherent to the use of mature cells are to be considered. First of all, mature cells cultured in vitro over long periods present the risk of dedifferentiation^60^. Moreover, in allogeneic implantation models, they may induce a strong host immune response against the foreign graft. In order to allow advanced studies in the context of allogeneic implantations in immunocompetent animals, undifferentiated stem cells are more appropriate. BMNCs seem to be a potential suitable candidate for TEVG cellularization because of their differentiation potential into both types of vascular cells. However, BMNCs are a heterogenous population composed of a large mix of cells, among them EPCs, ECs, MSCs, hematopoietic stem cells, T cells, and B cells, which induce complex interactions with host cells that are difficult to fully decipher and understand. This excessive heterogeneity could be a real obstacle for potential clinical applications where total control of the graft production is required, as well as a complete understanding of graft–host interactions. Several homogeneous stem cells populations possess the same differentiation potential as MDSCs or MSCs. Only a few studies on MDSCs are found in the literature compared to MSCs, which seem to be more appropriate for TEVG production. Indeed, their use could be more relevant since they have a homogeneous population, having adhesion capacity and differentiation potentials into both SMCs and ECs. Moreover, these cells are easily harvested from different tissues and are known to have important immunomodulatory properties which can be relevant to modulate the host immune system and avoid the graft rejection in the case of allogeneic cells^61^.

VTE combines cells, biomaterials and a suitable microenvironment for the production of small-caliber TEVG and aims to optimize the graft cellularization together with the biomechanical signals and scaffold structure. As described above, many studies have proposed the cellularization of a scaffold which will then be matured and implanted. Besides, other teams have developed innovative approaches to address several problems related to the TEVG production time and storage. The major challenge with cellularized TEVG is to find a suitable system and the combination of appropriate conditions for their storage without affecting the cell viability or the scaffold structure. Innovative cell-mediated production approaches concern the “Off-the-Shelf” and “Precast” grafts.

#### Off-the-shelf graft

Several teams are trying to produce a “ready-to-use” and storable graft that can be implanted in any patient, i.e., a “universal” or “off-the-shelf” graft. In order to propose such a graft, Tranquillo’s team has developed a decellularized graft originally composed by synthetic ECM^62^. They evaluated this original approach in several in vivo bypass models as a femoral bypass in sheep for 24 weeks^63^ and more recently as an arteriovenous bypass in baboons for up to 6 months^64^. In the last study, the graft was manufactured by seeding commercial human dermal fibroblasts into a bovine fibrinogen solution. The cell suspension was then injected into a tubular mold carrying a glass mandrel. Grafts were then cultured for 2 weeks and matured in a bioreactor for 3 supplementary weeks^62^. After maturation, grafts were decellularized and then stored in PBS at 4 °C. Grafts implanted as arteriovenous fistulas in baboons were evaluated by ultrasound assessments at 1, 2, 3, and 6 months, then scanned by angiography at the end of the study, just prior to collection. Graft patency rates were estimated at 83% at 3 months and 60% at 6 months. Moreover, an increase of the graft diameter was observed. This was probably induced by a remodeling of the graft tissue by vimentin and/or α-SMA positive cells and the colonization of the luminal surface by CD31 positive cells. However, some early thrombosis problems have been detected, probably due to the implantation site which has been changed to address these events in eight baboons. This led to a considerable reduction of the cohort. Results are promising and are similar to actual clinical solutions for arteriovenous fistula based on an ePTFE substitute. This model, although encouraging, is not representative of the small-diameter vessel bypass clinical situations, where grafts are subjected to challenging conditions like the blood pressure, the resistance to sutures and mechanical strains.

#### “Precast graft”

The use of autologous cells is limited by the lengthy manufacturing time, which may be incompatible with the clinical situation of the patient. In order to reduce the production delay, Quint et al.^18^ have proposed a storable decellularized graft which can be recellularized with autologous cells. Here, the graft was tested in a porcine carotid replacement model for a period of 30 days. A 4 mm diameter PGA mesh scaffold was seeded with a suspension of porcine aortic SMCs for 10 weeks under pulsatile conditions. The graft was then decellularized. Two cells sources were studied for the recellularization, either autologous ECs or EPCs, which were seeded onto the graft under pulsatile and rotational conditions for 2 h, followed by 2 h of static culture. The graft was then preconditioned in a bioreactor under a progressively increasing shear stress for 4–5 days. Three EPC-seeded grafts and two EC-seeded grafts were implanted and compared with a control group of three unseeded grafts and another control group consisting of 8 internal jugular veins. All the seeded grafts were still patent at 30 days post implantation compared to three control grafts (0/3 unseeded graft and 3/8 internal jugular veins). Unseeded grafts implantations have confirmed that the recellularized “precast graft” did not induce substantial inflammation. Moreover, a slow host cell colonization of the seeded graft matrix was highlighted by the presence of α-actin positive cells into the graft wall. In addition, tissue remodeling was also observed on the luminal surface where seeded cells were partially replaced by host ECs. Although the “precast graft” innovative approach seems to reduce the time required for the TEVG development, there is still a considerable amount of time for the patient autologous cell culture, plus 6 days of seeding and preconditioning. Moreover, the authors did not set up and define the storage conditions of their TEVG, which is one of the main criteria of this promising approach.

A relatively similar approach is reported by Dahl et al.^65^ by seeding a PGA scaffold with allogeneic canine SMCs (harvested from carotid and femoral arteries) for 7 to 10 days, followed by a decellularization step. The decellularized matrix was then washed and stored at 4 °C. The endothelialization step was performed by a fibronectin coating of the luminal surface of the TEVG followed by an autologous EC seeding under rotational conditions. The graft was then matured before implantation, by an increased shear stress preconditioning in a bioreactor for 35 h. The potential alterations of the grafts due to the storage conditions have been studied by mechanical characterizations, before and after a period of 12 months storage in PBS at 4 °C. After this storage period, no significant change was observed for burst pressure, compliance or suture strength. Eight grafts have been tested in vivo in dog models as follows: five in a carotid bypass model for up to 12 months and 3 grafts in a coronary bypass model for up to 1 month. During the study, two animals died with a patent graft and only one graft occluded at 1 week. Despite the histological analysis showing an incomplete EC coverage of the grafts luminal surface, 5 out of 6 grafts remained patent until the end of the study. Patent grafts showed a considerable remodeling, notably by α-SMA cells presumably derived from adjacent native vasculature.

## Clinical trials

This part of the review focuses on TEVGs with an inner diameter smaller than 6 mm that have progressed beyond the preclinical studies to phase I clinical trials and discusses the reported results as well as the encountered obstacles. Several medium (6–8 mm) and large (>8 mm)-diameter TEVGs have reached clinical trials such as the one of 8 mm of diameter developed by Olausson et al. consisting on a decellularized iliac artery seeded with autologous ECs and SMCs^66^, and the graft of 18 mm of diameter produced by Shin’oka et al. by autologous mononuclear BMCs seeded on a polymer tube^67^. Nevertheless, due to their specific clinical contexts, different from the problematic of this review, they will be excluded from this part of the review.

The most commonly used model in clinical trials is the arteriovenous fistula model, which allows the graft to be evaluated under nonlethal conditions. Moreover, this is a clinical situation in which TEVG would be of a great interest since the substitutes currently used are expanded polytetrafluoroethylene (ePTFE). In fact, ePTFE have a limited lifespan due to the damage of the graft wall by numerous punctures, thrombosis, infections and intimal hyperplasia in the long term^68^. However, the mechanical behavior, i.e., the blood pressure applied to TEVG in an arteriovenous shunt model, is much less compared to that found in other potential applications of the graft in blood vessel bypass grafting models, which makes it difficult to evaluate its effectiveness and performance under more challenging conditions.

### Endothelialized synthetic graft

Following in vivo preclinical studies on dogs^69^ and nonhuman primates^70^, the first clinical trial performed with a TEVG was between 1989 and 1991^71^. The study evaluated an endothelialized ePTFE graft in a femoropopliteal bypass model in 49 patients, in whom saphenous vein bypass was not applicable^71^. ECs were harvested from the right external jugular vein. An ePTFE graft with a diameter of 6 mm and a length of 60 cm was precoated with fibrin glue and seeded with autologous ECs, by filling the lumen with the cell suspension under rotation for 3 h. Then, the graft was implanted after 11.6 ± 3.0 days of a maturation period. Graft patency was determined by duplex sonography and Doppler ultrasonography after 9 days, 3 months, 6 months and 1 year of implantation. After 32 months, patency rates were estimated at 84.7% for endothelialized grafts vs. 55.4% for control non-endothelialized grafts. This endothelialized ePTFE TEVG was then included in a more generalized study involving 310 patients over a period of 15 years. Patients were divided into two groups, one group having a TEVG with a diameter of 6 mm, the other receiving a 7 mm diameter graft. The 6 mm diameter graft group has shown patency rates of 62% at 5 years and 55% at 10 years, vs. 78% and 71%, respectively, for the 7 mm diameter group^72^. Histological analysis of occluded grafts suggested that occlusions were not induced by an endothelialization defect but by large inflammatory foci located under the endothelium, causing a hyperplastic narrowing. Overall, the study demonstrates the interest of the integration of a VTE procedure as a routine therapy and confirms the benefit of autologous endothelialization of ePTFE grafts in clinical cases of infrainguinal bypass where no autologous vein is available. Major limitations of this graft include the use of an ePTFE synthetic scaffold that forms a static structure, preventing any possibility of growth and thereby excluding the graft use in a pediatric context. Moreover, the cellularization by autologous ECs implies several inconveniencies, such as the invasive collection of cells and the long-term in vitro culture that may increase the risk of contamination and loss of EC phenotype^73^.

### The Humacyte graft

Before performing clinical trials with the “Humacyte graft”, which is a complete “off the shelf” small-caliber graft, Niklason and her team tested a preliminary version of their TEVG using harvested human aorta SMCs. This graft has been evaluated in a baboon model of arteriovenous bypass for 1 to 6 months and has presented a patency rate of 88%^65^.

This Humacyte graft, a “human acellular vessel” (HAV), was produced by seeding human SMCs onto a biodegradable polymer scaffold (PGA) of 32 to 45 cm in length, for 8 weeks. The construction was matured in a bioreactor, applying pulsatile cyclic distension to allow the expansion of cells and the synthesis of their own ECM. The graft was then decellularized in order to obtain a tube of secreted ECM composed of different matrix proteins, such as collagens (I and III), fibronectin and vitronectin.

In 2014, enrollment of phase II trials (USA and Poland) was completed and 60 patients with end-stage renal disease (not candidates for fistula) had been grafted with the HAV “Humacyte graft” as an arteriovenous bypass for a period of 16 or 24 months^74^. The patency rates were checked by intraoperative ultrasound or Doppler and were valued at 63% and 28% at 6 and 12 months, respectively, (mostly due to thrombosis). The graft diameter increased during the implantation and reached 8.63 mm. During the clinical trial, some grafts at 16 to 55 weeks were collected after surgical revision procedures. Histological analysis revealed that at 16 weeks, the region near the vein anastomosis was colonized by myocytic cells expressing CD68 and α-SMA conjointly or separately, including myofibroblasts from surrounding tissue. The endothelialization of the graft was demonstrated by the detection of cells expressing CD31, VE-Cadherin, and eNOS at the luminal surface. At 46 weeks, the presence of these cells in the middle of the collected grafts seems to confirm that this phenomenon occurs from circulating blood and not from native adjacent vasculature. These two clinical studies have been extended to include 240 patients with a follow-up until 200 weeks^75^. Further histopathological analyses were conducted on collected grafts at different implantation times during the study to evaluate the HAV in vivo remodeling. These studies have revealed a tissue remodeling similar to that observed during embryonic vasculogenesis, which was characterized by the presence of Nestin+/CD34+ cells in the vascular wall at early stages. The number of these cells had decreased over time during implantation. In conclusion, the permeability results obtained with the “Humacyte” graft are substantially similar to those obtained with endothelialized ePTFE grafts. Despite its mechanical characteristics, similar or even superior to those of mammary arteries or saphenous veins, it suffers from the same shortcomings as synthetic grafts regarding repeated punctures. Indeed, several pseudoaneurysms were mentioned during these studies which are well-known complications of dialysis access with fistula or grafts.

### The Lifeline graft

L’Heureux and his team have developed an allogeneic small vascular graft called the “Lifeline graft” through “Sheet-Based Tissue Engineering” (SBTE). The TEVG was developed as follows: autologous fibroblasts were cultured and expanded, then the sheet of fibroblasts was decellularized (by dehydratation) and rolled up around a tube for 10 weeks, in order to obtain a homogenous cylinder. Another sheet of autologous fibroblasts was then used to cover the cylinder and the graft was kept in culture for a further 10 weeks. Autologous ECs were seeded into the graft several days before implantation (3–7 days), during which the graft was submitted to an increasing pulsatile flow in a bioreactor^76,77^. This graft was clinically tested in an arteriovenous shunt model in 10 end-stage renal disease patients. Patency rates were 78% and 60% at 1 and 6 months, respectively. The principal inconvenience of this autologous graft is the production time which reaches up to 26 weeks. Considering this limitation, the authors have recently proposed a SBTE graft with a shorter production time, manufactured with only allogeneic fibroblasts without ECs at the luminal surface^78^. This allogeneic graft was tested in the same model of hemodialysis access in three patients. Despite the nuclear debris that remained in the graft’s outer layer after decellularization, this study has shown that the Lifeline allogeneic graft did not induce deleterious immune responses. Grafts were implanted for up to 11 months, during which no aneurysms were detected, however surgical interventions were required to ensure secondary patency (2/3) and only one graft was patent for 7 months. These disappointing results could be due to an incomplete endothelialization of the graft which could have more severe consequences in coronary or peripheric bypass models. Currently, Magnan et al. are working on a new TEVG manufactured with a “textile approach”, also based on an ECM fibroblast sheet which is transformed into ribbons^79^. These ribbons are actually threads used to produce a TEVG of a diameter of 4.2 mm which is resistant to burst pressure. The first study on its immunogenicity and efficacy was carried out in a sheep carotid bypass model for 1 day and did not induce any complications.

## Conclusion

Currently, vascular replacement techniques using synthetic substitutes are successfully used for the replacement of large and medium diameter arteries. However, these substitutes are not suitable for the replacement of small-diameter arteries (<6 mm), principally due to a compliance mismatch and a lack of endothelialization of their luminal surface, leading to thrombotic events. Thus, one of the greatest challenges in VTE is to develop and propose to cardiovascular surgeons an effective endothelialized vascular prosthesis able to replace small-diameter arteries.

Since the first TEVG produced in 1986, the field of VTE seems devoted to a strong development and has never stopped to evolve, in order to create the “gold standard”, “readily available”, and “off-the-shelf” TEVG.

Recellularization of TEVG is one of the major factors that contributes to a better patency in a clinically relevant context^13^. As previously discussed, cells are widely used in the development of a small-caliber TEVG, in order to confer them with essential capacities that determine their success, such as hemocompatibility, patency, contractility, remodeling, growth or robustness. However, it should be noted that the cell types used in the TEVG manufacturing protocols are highly variable and can be from xenogeneic, allogeneic or autologous sources (summarized in Table 1), depending on the context of use of the TEVG. Indeed, there is no standardization or recommendation for the “best” cell origin, type and phenotype, allowing the development of the “perfect” TEVG. Although several cell types appear to be suitable for use in VTE, MSCs seem to be a promising choice for several reasons, such as their differentiation potential into vascular cells, their relatively easy collection and their immunomodulatory properties, allowing them to avoid immune rejection after transplantation.

**Table 1:** Overview of in vivo studied small-diameter TEVG produced by using cells.

Authors	Year	Ref.	Scaffold	Cells	Autologous/allogeneic	Animal model	Implantation site
*Preclinical studies*							
Ma et al.	2017	^19^	Fetal pig decellularized aortae	ECs	Autologous	Dog	Carotid
Kaushal et al.	2001	^21^	Pig decellularized iliac artery	EPCs	Autologous	Sheep	Carotid
Tillman et al.	2012	^22^	Pig decellularized carotid artery	EPCs	Autologous	Ovine	Arteriovenous shunt
Neff et al.	2011	^23^	Pig decellularized carotid artery	ECs + SMCs	Autologous	Pig	Carotid
Koch et al.	2010	^24^	Fibrin scaffold supported by a poly(L/D)lactide 96/4 (P(L/D)LA 96/4) mesh	ECs + SMCs	Autologous	Sheep	Carotid
Ju et al.	2017	^25^	Electrospun bilayered scaffold	EPCs + SMCs	Autologous	Sheep	Carotid
McIlhenny et al.	2015	^28^	Human decellularized saphenous vein	ASCs --> ELCs	Autologous	Rabbit	Aortae
Watanabe et al.	2011	^32^	Silicone rod	Biotube	Autologous	Rabbit	Carotid
Rothuizen et al.	2016	^34^	Polymer rod	Biotube	Autologous	Pig	Carotid
Yamanami et al.	2019	^35^	Silicone rod	Biotube	Autologous	Dog	Aortae
Roh et al.	2010	^38^	Biodegradable PGA-P(CL/LA)	hBMMCs	Allogeneic	SCID/bg mice	Inferior vena cava
Fukunishi et al.	2018	^40^	PGA-PLCL	mouse BMMCs	Allogeneic	C57BL/6 mice	Inferior vena cava
Hashi et al.	2007	^44^	Electrospun nanofibrous membrane	hBM-MSCs	Allogeneic	Athymic rat	Carotid
Akentiew et al.	2019	^45^	Dip spinning + solution blow spining	hBM-MSCs	Allogeneic	Rabbit	Carotid
Nieponice et al.	2010	^53^	ES-TIPS-PEUU	rat MDSCs	Allogeneic	Rat	Aortae
Hibino et al.	2012	^55^	PGA-P (CL/LA)	iPSCs --> ECs + SMCs	Allogeneic	SCID/bg mice	Inferior vena cava
Wang et al.	2014	^56^	PLLA	iPSCs --> SMCs	Allogeneic	Nude mice	Subcutaneous pocket
Itoh et al.	2015	^59^	Multicell spheroid approach	HUVECs + hSMcs + hFibroblasts	Allogeneic	Nude rat	Aortae
Syedain et al.	2017	^64^	Bovine fibrinogen solution	hFibroblast	Allogeneic	Baboon	Arteriovenous shunt
Quint et al.	2011	^18^	PGA mesh + porcine SMCs matrix	EPCs or ECs	Autologous	Pig	Internal jugular vein
Dahl et al.	2011	^65^	PGA scaffold + canine SMCs matrix	ECs	Autologous	Dog	Carotid + coronary bypass
*Clinical trials*							
Zilla et al.	1994	^71^	PTFE scaffold	ECs	Autologous	Human	Femoropopliteal bypass
Lawson et al.	2016	^74^	PGA scaffold + SMCs matrix	Decellularized		Human	Arteriovenous shunt
McAllister et al.	2009	^77^	Autologous fibroblast matrix	ECs	Autologous	Human	Arteriovenous shunt
Wystrychowski et al.	2014	^78^	Allogeneic fibroblast matrix	Decellularized		Human	Arteriovenous shunt

*ECs* Endothelial cells, *EPCs* endothelial progenitor cells, *SMCs* smooth muscle cells, *ASCs* adipose stem cells, *ELCs* endothelial-like cells, *hBMMCs* human bone marrow mononuclear cells, *hBM-MSCs* human bone marrow mesenchymal stem cells, *MDSCs* muscle derived stem cells, *iPSCs* induced pluripotent stem cells, *HUVECs* human umbilical vein endothelial cells, *TEVG* tissue-engineered vascular graft.

When using autologous cells, the cellularization of TEVG involves several issues, such as the very long production time, high manufacturing costs, and the storage conditions for allogeneic grafts. The storage factor can be addressed by a decellularization step after ECM production. Grafts tested in the last two described clinical trials were developed according to this method and appear to be efficient as arteriovenous shunts. However, the use of these TEVGs in other arterial bypass contexts, such as coronary or femoral arteries bypasses, have still not been evaluated, even though these are among the most challenging situations for demonstrating the performance of a vascular graft.

In addition, grafting technology is in constant progress. In fact, robotic surgery is potentially the most advanced medical technology and has been well documented in recent years^80,81^. In the long term, minimally-invasive robotic surgery should be accessible and performed for bypass surgeries on a daily basis. Thus, the development of a “gold standard” TEVG could supply a bank of ready-to-use grafts that would be available to cardiovascular surgeons.

In conclusion, our review shows the large heterogeneity of data produced in the study of cellularized small-diameter TEVG. This reflects the important need of small-diameter grafts and the considerable progress made in the VTE field in order to address this issue. However, this huge amount of data complicates the TEVG efficiency evaluation and the choice of the best development approach. There is an urgent need for standard guidelines for developing and testing TEVG in large animal models, at least regarding the cell type used for the recellularization. This would allow a proper comparison of the different strategies for the production of vascular grafts. The journey for finding the “gold standard” TEVG is on track to succeed, but there is still a long way to go for both research and clinical applications in the VTE field.
